# Autophagy inhibits chemotherapy-induced apoptosis through downregulating Bad and Bim in hepatocellular carcinoma cells

**DOI:** 10.1038/srep05382

**Published:** 2014-06-20

**Authors:** Yan Zhou, Kai Sun, Yi Ma, Haozheng Yang, Yuanliang Zhang, Xianming Kong, Lixin Wei

**Affiliations:** 1 Medical Sciences Research Center, Ren Ji Hospital, School of Medicine, Shanghai Jiao Tong University, Shanghai 200127, China; 2 Tumor Immunology and Gene Therapy Center, Eastern Hepatobiliary Surgery Hospital, The Second Military Medical University, Shanghai 200438, China; 3 Department of Biobank, Ren Ji Hospital, School of Medicine, Shanghai Jiao Tong University, Shanghai 200127, China; 4 Shanghai Institute of Hematology, Ruijin Hospital, School of Medicine, Shanghai Jiaotong University, Shanghai 200025, China

## Abstract

The tumor microenvironment, including ischemia, has been increasingly recognized as a critical factor in the process of tumor development. Hypoxia and nutrient deficiency resulting from ischemia widely exist in solid tumors. Recent studies have shown that hypoxia and nutrient deficiency contribute to chemoresistance by inducing autophagy, but the underlying mechanism remains unknown. This study aimed to explore the role of autophagy induced by low glucose and hypoxia (LH) in the chemoresistance of hepatocellular carcinoma cells. Our results demonstrated that LH induced autophagy and downregulated Bad and Bim in hepatocellular carcinoma cells. The inhibition of autophagy reversed the reduction of these pro-apoptotic factors during the LH treatment. Furthermore, Bad and Bim were also significantly downregulated by autophagy during the process that LH promoted the chemoresistance of hepatocellular carcinoma cells. In addition, RNAi or the overexpression of Bad and Bim can significantly reduce or increase chemotherapy-induced cell death, respectively. Taken together, these data indicate that the downregulation of Bad and Bim plays a significant role in the autophagy-induced chemoresistance of hepatocellular carcinoma cells.

Hepatocellular carcinoma (HCC) is one of the most common malignancies and is a leading cause of cancer-related mortality^1^. Surgery is the treatment that offers the greatest potential for a cure, but most patients have unresectable disease at presentation^2^. Other treatments such as chemotherapy are also widely used, especially for HCCs at an advanced stage. However, conventional systemic chemotherapy options have typically yielded poor outcomes for these patients. The tumor microenvironment, including ischemia, has been increasingly recognized as a critical factor in the process of tumor development^3^. Hypoxia and nutrient deficiency resulting from ischemia widely exist in solid tumors; however, cancer cells can survive in such an environment and continuously proliferate^4^,^5^. Recent studies have shown that autophagy plays an important role in protecting cancer cells that are subjected to hypoxia and nutrient deficiency^6^,^7^.

Autophagy is a conserved pathway crucial for development, differentiation, survival, and homeostasis^8^,^9^. The role of autophagy in cancer has been increasingly highlighted during the last decade. Autophagy is thought to be a predominant cell survival mechanism that is linked to a variety of physiological processes, such as aging, degenerative processes and nutrient starvation^10^. Increasing evidence shows that autophagy causes cell resistance to antineoplastic therapies. In these situations, the inhibition of autophagy may be a good therapeutic strategy^11^, and several inhibitors have been used, such as 3-methyladenine (3-MA)^12^, bafilomycin A1^13^ and chloroquine(CQ), and CQ is currently being used in a clinical trial^14^. 3-MA is an inhibitor of PI3K and inhibits autophagosome formation; CQ can inactivate lysosomal hydrolases by inhibiting lysosomal acidification, thereby restraining autophagy flux^15^,^16^.

Recent studies have showen that autophagy decreases the sensitivity of cancer cells to chemotherapeutic agents by affecting their apoptotic potential^17^,^18^,^19^. In this study, we detected autophagy under conditions of low glucose and hypoxia (LH) and investigated the effects of LH on autophagy in HCC cells exposed to chemotherapeutic agents. In addition, we examined whether the inhibition of autophagy enhanced the chemotherapy-induced apoptosis of HCC cells.

## Results

### Low glucose and hypoxia induce autophagy in HCC cells

**Figure 1:**
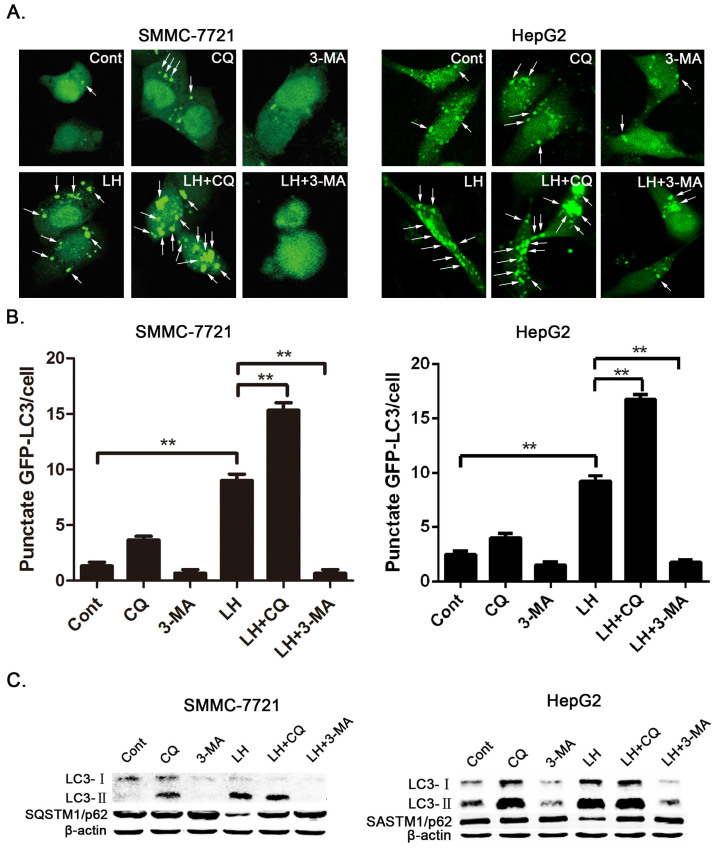
Autophagy was induced under conditions of LH and inhibited by CQ and 3-MA in HCC cells. (A–C). SMMC-7721 and HepG2 cells were directly incubated under normal or LH conditions for 8 h or incubated under LH conditions in the presence or absence of CQ or 3-MA for 8 h. (A). The cells were transfected with GFP-tagged LC3 and observed with fluorescence microscopy. Arrows show the punctate GFP-LC3 fluorescence in the cytoplasm. (B). The number of punctate GFP-LC3 in each SMMC-7721 or HepG2 cell was counted, and at least 100 cells were included for each group. The data represent the mean ± SD based on three independent experiments. **p < 0.01. (C). The whole-cell lysates were subjected to western blot analysis as described in the ‘Materials and methods'section. The experiments were repeated at least three times.

The tumor microenvironment plays an important role in the chemoresistance of tumor cell. Hypoxia and nutrient deficiency are important characteristics of the tumor microenvironment. Increasing evidence shows that autophagy contributes to the chemoresistance in cancer cells. Therefore, we first determined whether LH can activate autophagy in HCC cells. We examined autophagy under conditions of LH with an expression vector encoding GFP-LC3, which is concentrated in autophagic vacuoles and results in punctate fluorescence within the cells. SMMC-7721 and HepG2 cells were transiently transfected with GFP-LC3 plasmids. Twenty-four hours after transfection, the cells were treated with autophagy inhibitors and incubated under normal or LH conditions. After 8 hours of treatment, the cells were observed under a fluorescence microscope, and the cells with GFP-LC3 puncta were counted. As shown in Figure 1, a higher percentage of cells with punctate LC3 fluorescence staining was observed in the cells under conditions of LH than in those under normoxic conditions. The data also showed that CQ or 3-MA effectively and dramatically inhibited the autophagy response induced by LH (Figure 1A and 1B). To confirm the level of autophagy with additional independent assays, we analyzed the protein expression levels of LC3 and SQSTM1/p62 with a western blot assay. LH resulted in a remarkable increase in the level of LC3-II and a decrease in SQSTM1/p62 levels in SMMC-7721 and HepG2 cells compared with cells grown under normal conditions (Figure 1C). In addition, we separately compared a low-glucose treatment and a hypoxia treatment with the LH treatment, and the results indicated that the induction of autophagy is primarily caused by the combination treatment (LH) (Supplementary Figure 1). The above results showed that autophagy was significantly activated in response to LH.

### Autophagy leads to the downregulation of Bad and Bim in HCC cells

**Figure 2:**
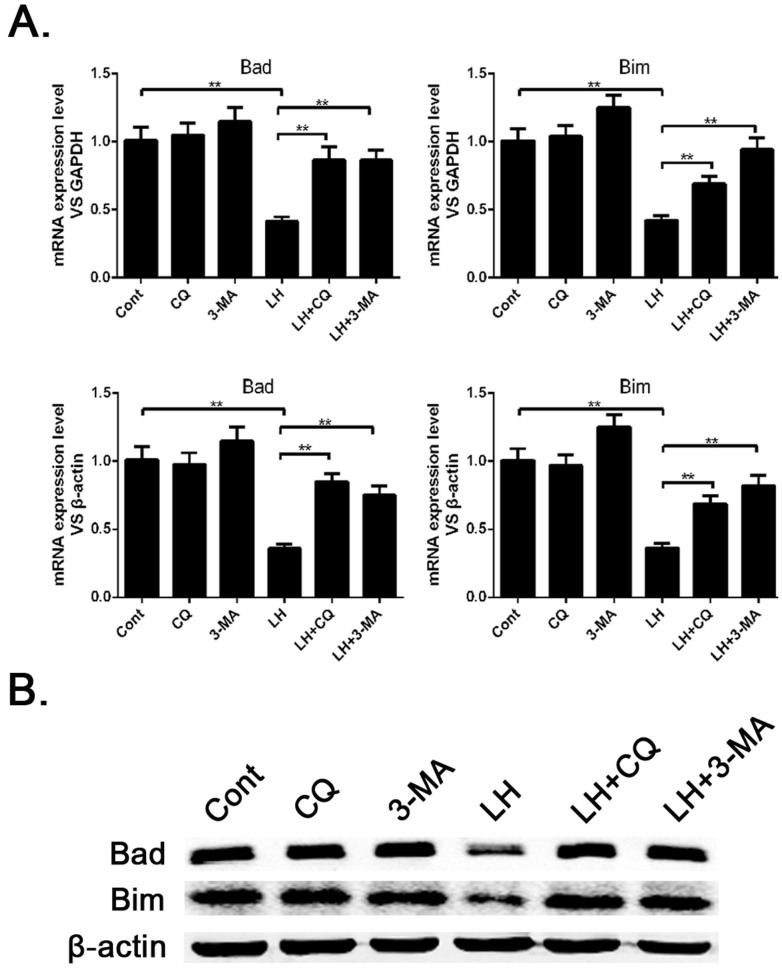
Autophagy induced by LH resulted in a decrease of pro-apoptotic proteins in the SMMC-7721 cells. (A). The mRNA levels of apoptotic genes, including Bad and Bim, in SMMC-7721 cells cultured under normal or LH conditions were detected by q-PCR. The reference genes were GAPDH and β-actin. The data represent the mean ± SD based on three independent experiments. ** p < 0.01. (B). The expression of pro-apoptotic proteins was analyzed by western blotting, and the experiments were repeated at least three times.

Autophagy has been shown to inhibit apoptosis in cancer cells. To further study how autophagy regulates cell survival, we decided to examine whether autophagy is involved in regulating pro-apoptotic factors in HCC cells. First we incubated SMMC-7721 cells under conditions of LH for 8 h and examined the levels of pro-apoptotic genes with a q-PCR method. We found a drastic decrease in the mRNA levels of some pro-apoptotic genes, including Bad and Bim, in the SMMC-7721 cells under LH conditions compared with the levels under normal conditions, while CQ or 3-MA, to some extent, attenuated the downregulation of these genes (Figure 2A). We then determined the expression levels of these pro-apoptotic proteins with western blot analysis, and the results were consistent with the q-PCR data (Figure 2B). These data suggest that LH-induced autophagy downregulates the expression levels of these pro-apoptotic proteins in SMMC-7721 cells but does not affect the phenotype of the apoptotic cells. The expression levels of Bad and Bim regulated by autophagy are independent of the apoptotic cell phenotype.

### Autophagy inhibits the chemotherapy-induced apoptosis of HCC cells

**Figure 3:**
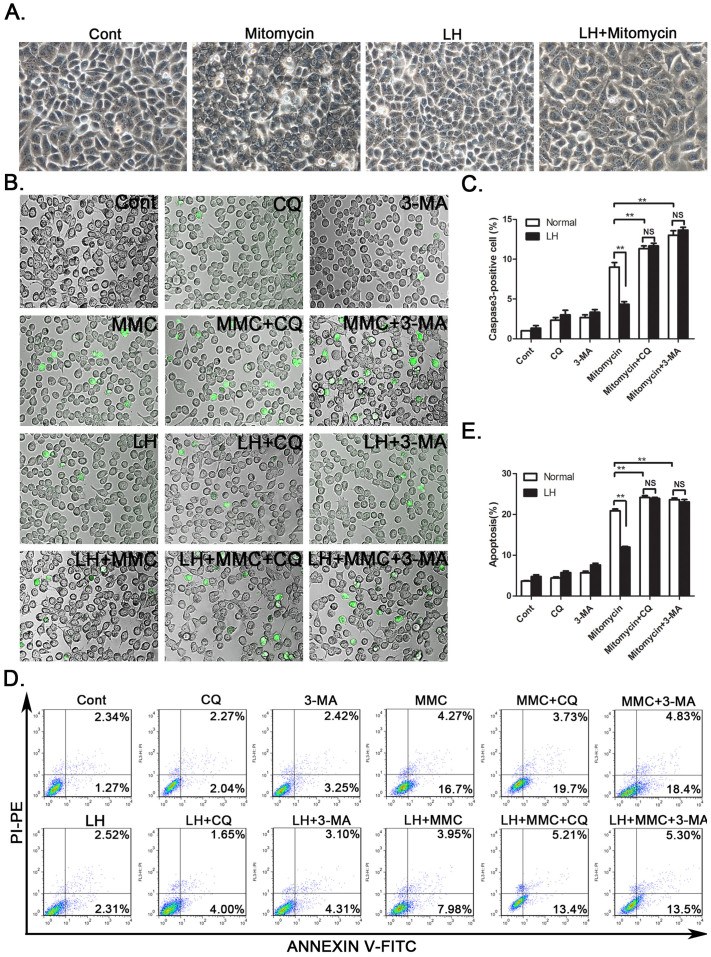
Chemotherapeutic agent-induced cell apoptosis was attenuated by autophagy in HCC cells. (A). SMMC-7721 cells were treated with mitomycin (2 μM) under LH conditions for 24 hours. The morphology of the cells was recorded with a light microscope. (B). The fluorescent microscopic images of the apoptotic cells were captured via caspase3 staining. The quantification of the capase3-positive cells was described (C). (D and E). Annexin V staining and FACS analysis were performed after the mitomycin treatment for 24 h under normal or LH conditions in SMMC-7721 cells; the percentage of annexin V^+^ cells represents the apoptotic cells. MMC: Mitomycin. The experiments were repeated at least three times. **p < 0.01; NS, no significance.

Next, we determined whether LH-induced autophagy contributes to the chemoresistance of HCC cells. SMMC-7721 cells were cultured under normal or LH conditions for 8 h in the presence or absence of mitomycin. Notably, mitomycin caused more significant morphological changes under normal conditions than under LH conditions. Many cells exhibited detachment, shrinkage and karyopyknosis under normal conditions (Figure 3A). Moreover, there were more caspase3-positive cells induced by mitomycin under normal conditions compared with the number observed under the LH conditions (Figure 3B and 3C). To confirm these results, we assayed cell apoptosis with flow cytometry. The data, as indicated by the annexin V-FITC assay, showed that approximately 20% of the cells underwent apoptosis with the mitomycin treatment under normal conditions, while only approximately 10% of the cells under the LH condition were apoptotic (Figure 3D and 3E). In contrast, CQ or 3-MA was found to distinctly inhibit the protective effect of autophagy (Figure 3B–3E). These results demonstrated that SMMC-7721 and HepG2 cells exposed to LH had enhanced resistance to chemotherapeutic agents due to the activation of autophagy.

### Inhibition of autophagy increases chemotherapy-induced cell death

**Figure 4:**
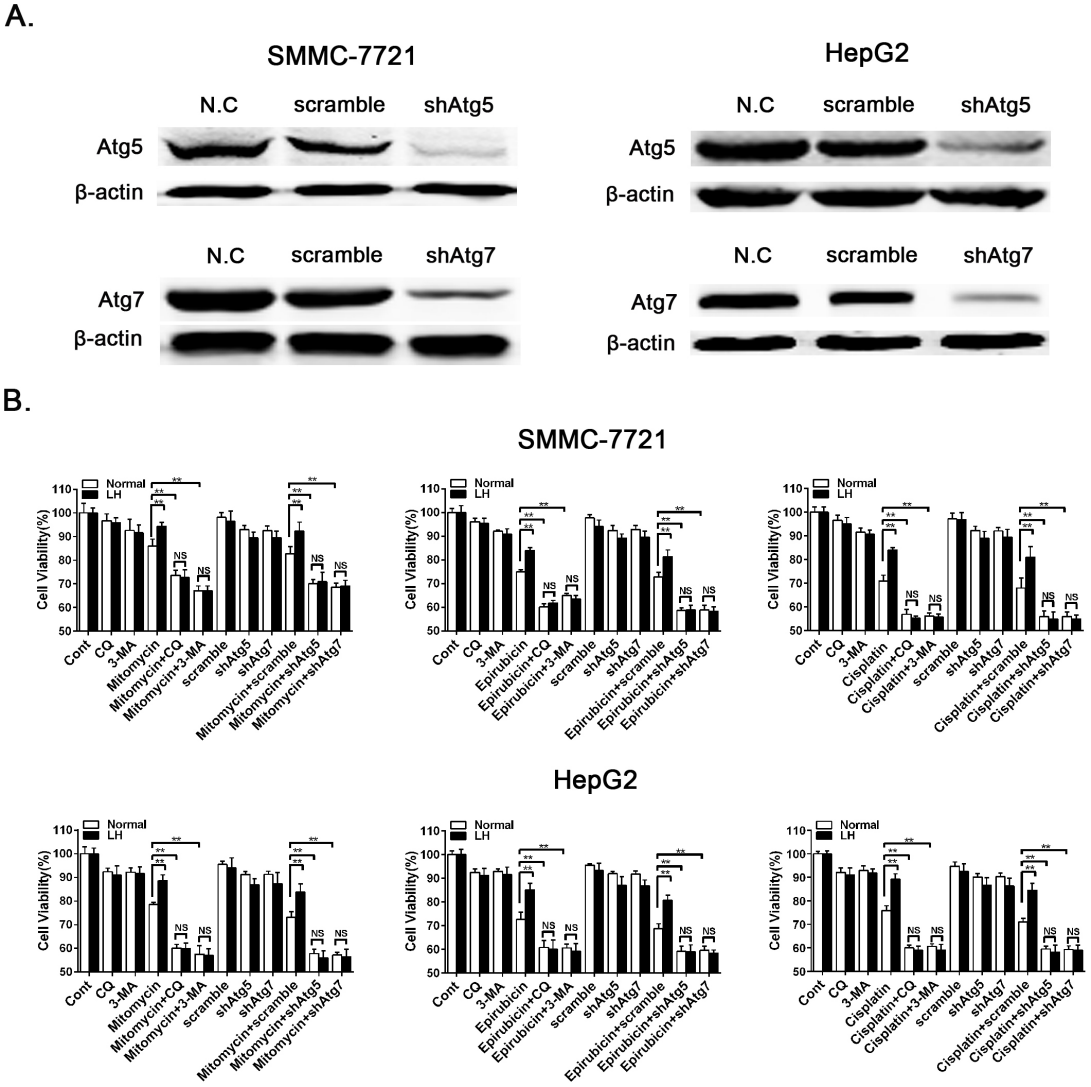
Inhibition of autophagy increased chemotherapeutic agent-induced cell death. (A). The knockdown efficiency of Atg5 and Atg7 in the SMMC-7721 and HepG2 cells were determined by western blot analysis, and the experiments were repeated at least three times. (B). SMMC-7721 and HepG2 cells were treated with mitomycin (2 μM), epirubicin (2 μM) or cisplatin (20 μM) under normal or LH conditions for 24 h. The cell viability was measured with the CCK8 assay. The data represent the mean ± SD based on six independent determinations. **p < 0.01; NS, no significance.

To further confirm that the inhibition of autophagy can significantly increase chemotherapy-induced cell death, we treated SMMC7721 and HepG2 cells with the autophagy inhibitors CQ or 3-MA and knocked down the essential autophagy genes Atg5 or Atg7. Atg5 and Atg7 were effectively knocked down with shRNA lentiviruses (Figure 4A). We then determined the cell viability with a CCK8 colorimetric assay after treatment with the chemotherapeutic agents, including mitomycin, epirubicin and cisplatin; the results were consistent with those from the cell apoptosis assay (Figure 4B). These data demonstrate that the inhibition of autophagy can clearly enhance the chemosensitivity of HCC cells.

### Autophagy contributes to the chemoresistance of HCC cells by downregulating Bad and Bim

**Figure 5:**
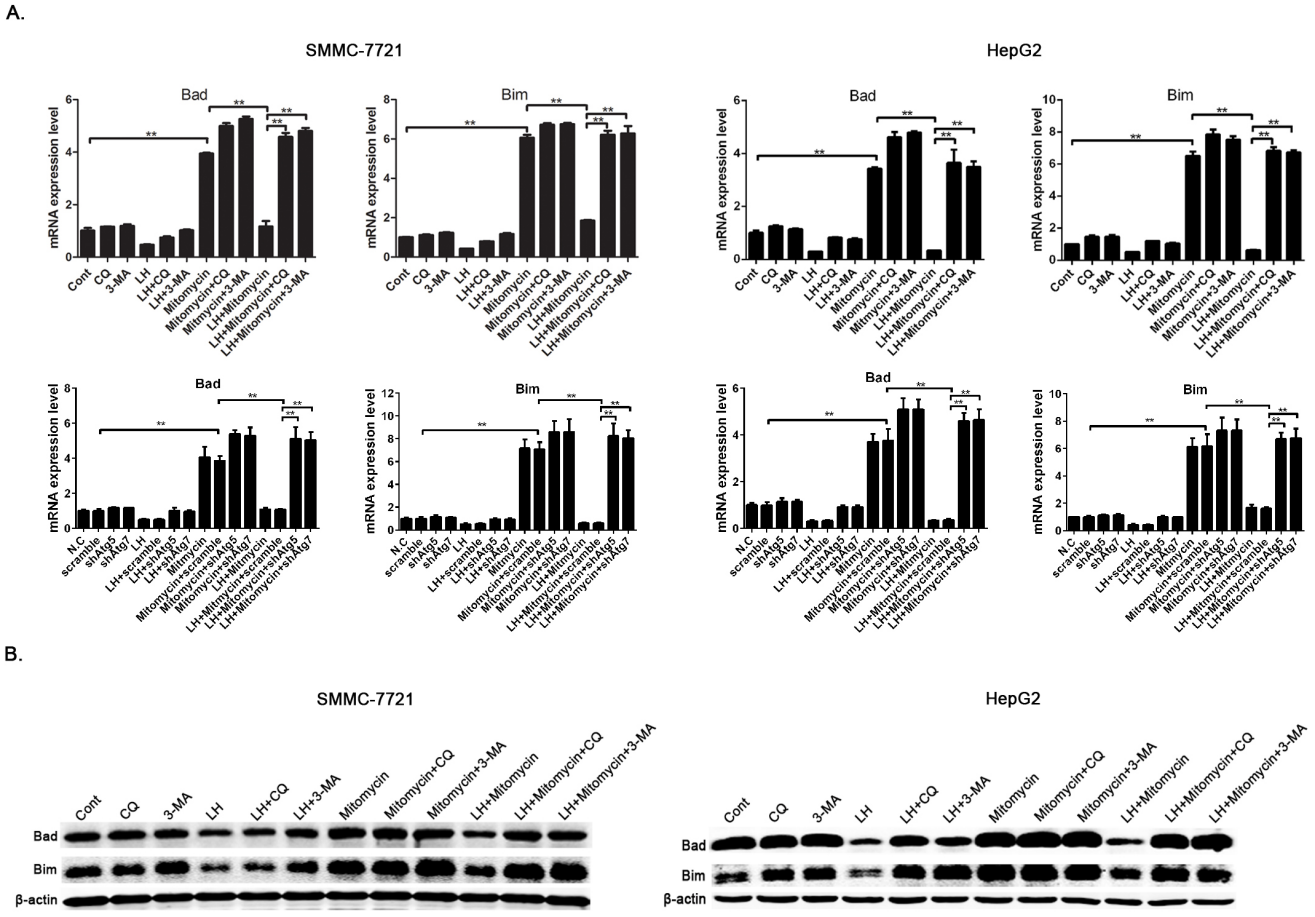
Autophagy leads to chemoresistance through the downregulation of Bad and Bim in HCC cells. (A). The mRNA levels of Bad and Bim in the SMMC-7721 and HepG2 cells, which were incubated under normal or LH conditions in the presence or absence of CQ or 3-MA or with the knock down of Atg5 or Atg7, were detected via q-PCR. The data represent the mean ± SD based on three independent experiments. ** p < 0.01. (B). The expression levels of the two pro-apoptotic proteins were analyzed by western blotting, and the experiments were repeated at least three times.

To determine whether autophagy inhibits chemotherapy-induced apoptosis by downregulating pro-apoptotic proteins, we compared the expression levels of Bad and Bim between normal and LH culture conditions in the presence of mitomycin. The results revealed that, after mitomycin treatment, the mRNA levels of Bad and Bim were downregulated when autophagy was induced compared with those observed when autophagy was not induced, and the autophagy inhibitors CQ or 3-MA or the knockdown of Atg5 or Atg7 can distinctly reverse this trend (Figure 5A). We also tested the protein levels of Bad and Bim with western blot analysis, and the results were consistent with the q-PCR data (Figure 5B). These results suggest that autophagy can enhance the chemoresistance of HCC cells through downregulating Bad and Bim and that autophagy inhibition can reverse the inhibitory effect of autophagy on the expression of these proteins, thereby restoring the chemosensitivity.

### RNAi or Overexpression of Bad and Bim increases or reduces chemotherapy-induced cell death, respectively

**Figure 6:**
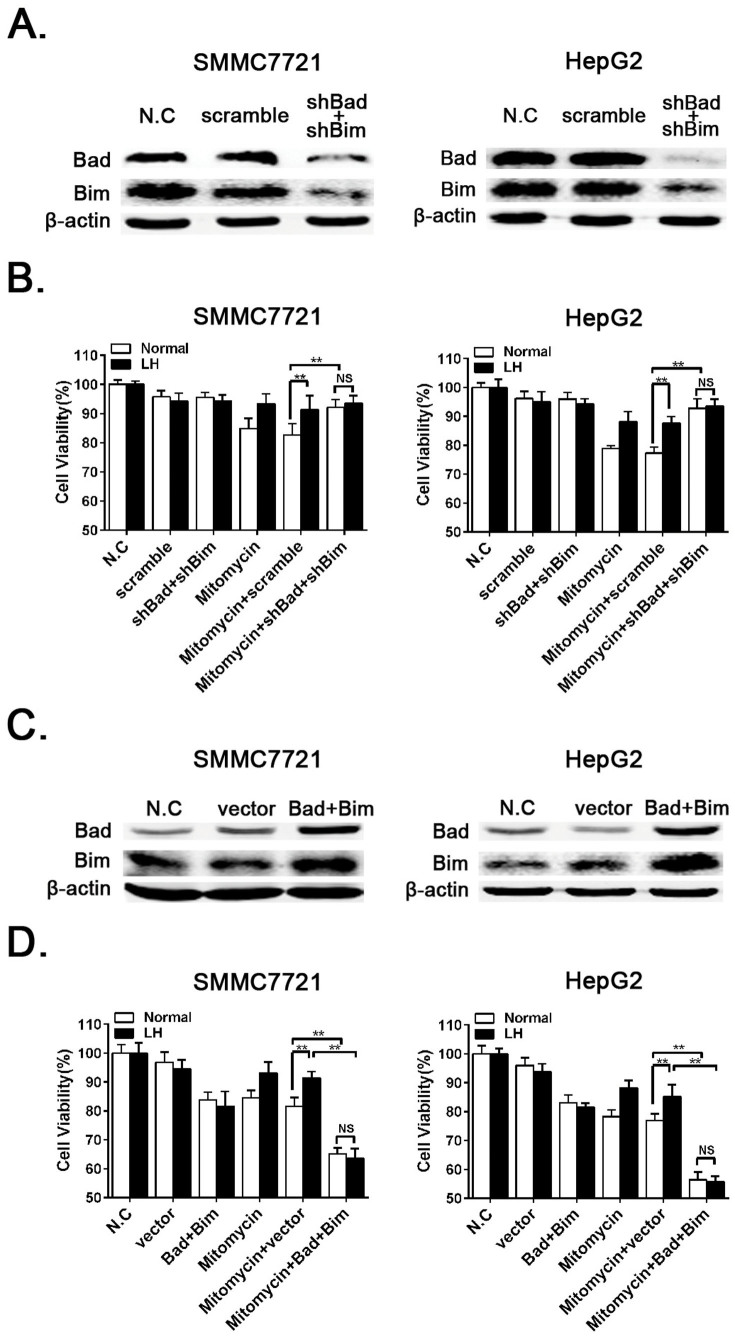
RNAi of Bad and Bim reduces chemotherapeutic agent-induced death, and autophagy-induced chemoresistance can be overcome by the overexpression of exogenous Bim and Bad. (A and C). The RNAi or overexpression of Bad and Bim in SMMC-7721 and HepG2 cells were verified by western blot analysis, and the experiments were repeated at least three times. (B and D). SMMC-7721 and HepG2 cells were treated with mitomycin (2 μM) under normal or LH conditions for 24 h with the RNAi or overexpression of Bad and Bim. The cell viability was measured with the CCK8 assay. The data represent the mean ± SD based on six independent determinations. **p < 0.01; NS, no significance.

To provide insight to the contributions of Bad and Bim to autophagy-mediated chemoresistance, we knocked down Bad and Bim with shRNA lentiviruses (Figure 6A). We then determined the cell viability with a CCK8 colorimetric assay, and the data showed that the RNAi of Bad and Bim clearly reduced chemotherapy-induced cell death (Figure 6B). To further determine the function of Bad and Bim in autophagy-induced chemoresistance, we utilized the overexpression of exogenous Bad and Bim in HCC cells to examine whether this overexpression can rescue autophagy-induced chemoresistance. First, Bad and Bim were successfully overexpressed via lentivirus infection (Figure 6C). We then assayed the cell viability, and the results showed that the overexpression of Bad and Bim increased mitomycin-induced cell death despite of the protective effect of LH-induced autophagy (Figure 6D). These results indicated that LH-induced autophagy enhanced cell survival via the downregulation of Bad and Bim.

## Discussion

HCC is one of the most common cancers worldwide. The failure of chemotherapy is often due to drug resistance. Improving chemosensitivity is necessary for tumor therapy in the clinic. Numerous studies have shown that autophagy may be activated under stress in various cancer cells and may play an important role in the processes of tumorigenesis and tumor development^20^. However, the underlying mechanism still requires clarification.

Some studies have reported that hypoxia and nutrient deficiency can activate autophagy^21^,^22^. In our study, the percentage of cells with punctate staining of GFP-LC3 significantly increased in response to LH. We therefore examined the effects of the autophagic inhibitors CQ and 3-MA on the occurrence of autophagy and found that either of these two inhibitors could effectively inhibit autophagy induced by LH in SMMC-7721 and HepG2 cells (Figure 1).

Several aspects of the biological role of autophagy are still unclear, and the relationship between apoptosis and autophagy, particularly in the liver, has yet to be thoroughly explored. With the confirmation of autophagy in SMMC-7721 and HepG2 cells under LH conditions, the relationship of autophagy to cell apoptosis deserves our attention. We found that the expression levels of the pro-apoptotic proteins Bad and Bim were downregulated in HCC cells under LH conditions compared with those in cells under normal conditions, while CQ or 3-MA, to some extent, could counteract this trend (Figure 2B). This result suggests that autophagy downregulates the expression of pro-apoptotic proteins. It is well known that autophagy is a proteolytic pathway, so how can this process downregulate the mRNA expression levels of Bad and Bim (Figure 2A)? Previous studies have shown that the transcriptional expression levels of Bad and Bim can be regulated by certain signaling pathways, such as those for JNK^23^ and TGF-β^24^. In addition, the relationship between JNK, TGF-β and autophagy has been widely reported^25^,^26^; therefore, some regulatory factors in the JNK or TGF-β pathways may be degraded by autophagy, which would cause the transcriptional downregulation of Bad and Bim.

Autophagy induced by hypoxia and nutrient deficiency is often correlated with a poor prognosis and high patient mortality rate, which is partly due to the resistance to chemotherapy^27^,^28^,^29^. Consistent with this concept, the results from the caspase3 staining and the flow cytometry and CCK-8 assays in this study showed that the autophagy induced by LH protected the HCC cells from mitomycin-, epirubicin- or cisplatin-induced apoptosis (Figure 3 and 4). These results indicate that autophagy may play a protective role in the process of chemotherapy-induced apoptosis, leading to the chemoresistance of the HCC cells. During the course of treatment with the chemotherapeutic agents, the inhibition of autophagy by CQ or 3-MA or the knockdown of Atg5 or Atg7 significantly enhanced the cytotoxic effects of the chemotherapeutic agents and increased the rate of apoptosis in SMMC-7721 cells. These results suggest that autophagy inhibitors may be used as novel sensitizers to improve the effects of chemotherapy drugs.

Many previous studies have reported that autophagy inhibits the apoptosis induced by chemotherapy drugs in cancer cells, but the underlying mechanisms have not been clearly elucidated. In our study, we demonstrated that LH-induced autophagy downregulated the expression of the pro-apoptotic proteins Bad and Bim in the presence of chemotherapeutic agents in the HCC cells, while CQ or 3-MA or the RNAi of Atg5 or Atg7 can counteract this trend (Figure 5). Bad and Bim are key proteins in the apoptotic pathway, and the downregulation of these factors plays an important role in autophagy-induced chemoresistance. The RNAi of Bad and Bim or overexpression of Bad and Bim can directly enhance or overcome autophagy-induced chemoresistance (Figure 6). These data reveal a novel mechanism for the downregulation of apoptosis-related signaling via autophagy.

In conclusion, our results demonstrate that the autophagy induced by LH inhibits chemotherapy-induced apoptosis by downregulating Bad and Bim in hepatocellular carcinoma cells. This finding not only deepens our understanding of the mechanisms involved in and the relationship between autophagy and apoptosis, but also aids the further study of the function of autophagy in cancer cells especially in HCC cells. We predict that targeting the autophagy and apoptosis pathways is a promising therapeutic strategy to enhance the effects of chemotherapy and improve the clinical outcomes in HCC patients.

## Methods

### Cell culture and reagents

The human HCC cell line SMMC-7721 and HepG2 were maintained in Dulbecco's modified Eagle's medium (high glucose or low glucose) (GIBCO, Life Technologies, Cat.11995-065/Cat.11885-084) supplemented with 10% fetal bovine serum (GIBCO, Life Technologies, Cat.10099-141), 100 units/ml penicillin, and 100 mg/ml streptomycin (GIBCO, Life Technologies, Cat.15140-122) in a humidified incubator with 95% air and 5% CO_2_ at 37°C. For the low glucose and hypoxia condition, the cells were cultured in Dulbecco's modified Eagle's medium (low glucose) (GIBCO, Life Technologies, Cat.11885-084) in a CO_2_ incubator (Thermo Scientific) maintained at 94% N_2_, 5% CO_2_ and 1% O_2_. CQ (Sigma-Aldrich, Cat. C26628) and 3-MA (Sigma-Aldrich, Cat. M9281) were used at 10 μM and 5 mM respectively.

### Transient transfection and identification of autophagy

A GFP-tagged LC3 expression vector has recently been utilized to detect autophagy. SMMC-7721 and HepG2 cells were seeded (2 × 10^5^ cells/well) into 12-well plates overnight. GFP-LC3-expressing plasmids were then transiently transfected into the cells with Lipofectamine™ 2000 (Invitrogen, Life Technologies, Cat.11668-019) according to the manufacturer's instructions. Twenty-four hours after the transfection, the cells were subjected to LH conditions for 8 h, and in some experiments, CQ and 3-MA were also added. At the end of the treatment, autophagy was detected by counting the percentage of cells with GFP-LC3 puncta with confocal microscopy (Leica SP5). A minimum of 200 cells per sample were counted, and each experiment was performed in triplicate.

### Western blot analysis

After the treatments, the SMMC-7721 and HepG2 cells were collected and lysed in protein extraction reagent (Thermo Scientific, Cat.78501) with 100× cocktail (Roche). Equal amounts of protein were separated by SDS-PAGE and transferred on to a nitrocellulose membrane (Whatman, Cat.10401396). After blocking (LI-COR, Inc., Cat.927-40000), the membrane was incubated with the primary rabbit monoclonal antibodies against Bad (Cell Signaling Technology, CST, Cat.9239), Bim (CST, Cat.2933), SQSTM1/p62 (CST, Cat.5114), Atg5 (CST, 8540), Atg7 (CST, 8558), and β-actin (HangZhou HuaAn Biotechnology Co., Ltd., China, R1207-1) and the primary rabbit polyclonal antibody against LC3B (Novus Biologicals, Inc., Cat.NB600-1384). After incubation with the secondary antibodies labled with fluorescence (LI-COR, Cat.926-32211), the proteins were visualized by fluorescence with an Infrared Imaging System (ODYSSEY). The LC3B, SQSTM1/p62, Bad, Bim, Atg5 and Atg7 protein expression levels were normalized with β-actin.

### RNA isolation, reverse transcription, and quantitative real-time PCR (q-PCR)

The total RNA isolation, cDNA synthesis and q-PCR were previously described^30^. Total RNA was extracted from the SMMC-7721 and HepG2 cells with TRIzol according to the manufacturer's instructions (Ambion, Life Technologies, Cat. 15596018). The RNA concentrations were determined with spectrophotometrically.

For the cDNA synthesis, 1 μg of RNA was reverse transcribed with the RT Reagent Kit with gDNA Eraser (TaKaRa, Cat.DRR047S). The reverse transcription reaction was completed at 37°C for 15 min and 85°C for 5 s. The cDNA was stored at −20°C.

The mRNA expression of the genes was analyzed with specific primers as follows:

5′-GAAGGTGAAGGTCGGAGTC-3′, 5′-GAAGATGGTGATGGGATTTC-3′ for GAPDH, and the length of this amplicon was 225 bp;

5′-CGGAGGATGAGTGACGAGTT-3′, 5′-GATGTGGAGCGAAGGTCACT-3′ for Bad, and the length of this amplicon was 180 bp;

5′-TAAGTTCTGAGTGTGACCGAGA-3′, 5′-GCTCTGTCTGTAGGGAGGTAGG-3′ for Bim, and the length of this amplicon was 96 bp.

The q-PCR was conducted in a Roche LightCycler480 using the SYBR Premix Ex Taq (TaKaRa, Cat. DRR420A). The 2-step q-PCR was as follows: denaturation at 95°C for 30 seconds, followed by 45 cycles of 95°C for 5 seconds and 60°C for 20 seconds. All of the tests were performed in triplicate. A no template control was used as the negative control for each run. The relative gene expression was calculated with using the ΔΔCt method, and GAPDH was used as the internal control.

### Cell apoptosis assays via caspase3 staining and flow cytometry

Cells (2 × 10^5^ cells/well) were seeded into 6-well plates overnight and then exposed to LH for 8 h. Then, we added mitomycin to the medium and continued to culture these cells for 16 h under LH conditions, and in some experiments, CQ (10 μM) and 3-MA (5 mM) were used to block autophagy. After these incubations, we replaced the medium with fresh medium or PBS containing 5 μM caspase3 substrate stock solution (NucView, Cat.30029) and incubated the cells at room temperature for 30 minutes. The cells were washed and then observed with fluorescence microscopy in PBS using filter sets for green fluorescence (excitation/emission: 485/515 nm). In addition, an annexin V-FITC assay was used to measure the apoptotic cells via flow cytometry according to the manufacturer's instructions (Nanjing Keygen Biotech, China, Cat.KGA108). Briefly, the cells were collected by trypsinization (without EDTA), washed twice with phosphate-buffered saline (PBS) and resuspended in 500 μl of 1× binding buffer containing 5 μl annexin V and 5 μl PI for 15 minutes at room temperature in the dark. After the incubation, at least 10^5^ cells were measured with a BD FACSCalibur flow cytometry (BD Biosciences). The results were presented as the percentage of apoptotic cells (annexin V positive).

### Lentivirus Infection

To generate plasmids for stable RNAi, oligonucleotides encoding shRNAs specific for Bad and Bim and a related scrambled sequence were cloned into the EcoRI and BamHI sites of the lentiviral vector pLSLG under the control of a constitutive U6 promoter, and this shRNA vector also encoded an EGFP protein. shRNA lentiviruses specific for Atg5 and Atg7 were purchased from Shanghai GeneChem. The following oligonucleotides were used for the shRNA experiments: Atg5: 5′-CCTTTCATTCAGAAGCTGTTT-3′; Atg7: 5′-CCAAGGTCAAAGGACGA AGAT-3′; Bad: 5′-AGAGGATCCGTGCTGTCTC-3′; Bim: 5′-CACCCACGAATGG TTATCTTA-3′; scrambled sequence: 5′-GACCGGATAGTAACCTTCA-3′.

To generate plasmids for stable overexpression, the Bad and Bim cDNAs were cloned into the lentiviral vector MigRI.

For lentiviral production, either the shRNA plasmids and the package plasmids (PMD2 and PSPAX) or the overexpression plasmids and the package plasmids (VSVG and gag-pol) were cotransfected into 293T cells using the calcium phosphate method. The supernatants were collected 48 h after the transfection, filtered through a 0.45-µm membrane and concentrated by ultracentrifugation. The SMMC7721 and HepG2 cells were then infected with lentiviral vecors for 4 hours, and after serial passaging, the GFP-positive cells were sorted for subsequent experiments.

### Cell Counting Kit-8

The cell viability assays were performed with the Cell Counting Kit-8 (DOJINDO, Japan). Cells (7 × 10^3^ cells/well) were first seeded into 96-well plates, incubated overnight and then exposed to LH for 8 h. After that, we added the indicated chemotherapeutic agents to the medium and continued to culture the cells for 16 h under LH conditions. In some experiments, CQ (10 μM) and 3-MA (5 mM) were used to block autophagy. As soon as the treatment was completed, 10 μl of the Cell Counting Kit-8 reagent was added to each well, and the cells were incubated for 2 h at 37°C. Finally, the spectrophotometric absorbance of each sample was measured with a microplate reader (Synergy HT, Bio-Tek) at 450 nm; based these readings, the percentage of surviving cells in each treated group was plotted. All of the experiments were carried out with six replicates.

### Statistical analysis

All of the experiments were repeated at least three times. The data were presented as the mean ± SD (standard deviation). Student's t test was used to compare the means. The two-tailed p-value for statistical significance was defined as p< 0.01. The statistical analyses were performed using the GraphPad Prism 5.0 software (Graphpad Software, San Diego, CA).

## Author Contributions

Y.Z., K.S., Y.M., H.Z.Y. and Y.L.Z. participated in the design and performance of this study. Y.Z. carried out cell culture and Western Blot analysis. Y.Z. and K.S. performed cell apoptosis analysis. Y.Z. and H.Z.Y. carried out confocal microscope experiment. L.X.W. and X.M.K. conceived of the study, supervised the work, analyzed data and provided financial support. The manuscript was drafted by Y.Z., and reviewed by all authors. All authors approved the final version of the manuscript to be published.

## Supplementary Material

Supplementary Information Dataset 1

## References

[b1] Befeler A. S. & Di Bisceglie A. M. Hepatocellular carcinoma: diagnosis and treatment. Gastroenterology 122, 1609–1619 (2002). 12016426 10.1053/gast.2002.33411

[b2] Raza A. & Sood G. K. Hepatocellular carcinoma review: Current treatment, and evidence-based medicine. World J Gastroenterol. 20, 4115–4127, 10.3748/wjg.v20.i15.4115 (2014). 24764650 10.3748/wjg.v20.i15.4115 PMC3989948

[b3] Swartz M. A. *et al.* Tumor microenvironment complexity: emerging roles in cancer therapy. Cancer Res. 72, 2473–2480, 10.1158/0008-5472.CAN-12-0122 (2012). 22414581 10.1158/0008-5472.CAN-12-0122 PMC3653596

[b4] Song J. *et al.* Autophagy in hypoxia protects cancer cells against apoptosis induced by nutrient deprivation through a Beclin1-dependent way in hepatocellular carcinoma. J Cell Biochem. 112, 3406–3420, 10.1002/jcb.23274 (2011). 21769915 10.1002/jcb.23274

[b5] Sato K. *et al.* Autophagy is activated in colorectal cancer cells and contributes to the tolerance to nutrient deprivation. Cancer Res. 67, 9677–9684, 10.1158/0008-5472.CAN-07-1462 (2007). 17942897 10.1158/0008-5472.CAN-07-1462

[b6] Yang Z. & Klionsky D. J. An overview of the molecular mechanism of autophagy. Curr Top Microbiol Immunol. 335, 1–32, 10.1007/978-3-642-00302-8_1 (2009). 19802558 10.1007/978-3-642-00302-8_1 PMC2832191

[b7] Song Y. J. *et al.* Autophagy contributes to the survival of CD133+ liver cancer stem cells in the hypoxic and nutrient-deprived tumor microenvironment. Cancer Lett. 339, 70–81, 10.1016/j.canlet.2013.07.021 (2013). 23879969 10.1016/j.canlet.2013.07.021

[b8] Degenhardt K. *et al.* Autophagy promotes tumor cell survival and restricts necrosis, inflammation, and tumorigenesis. Cancer Cell 10, 51–64, 10.1016/j.ccr.2006.06.001 (2006). 16843265 10.1016/j.ccr.2006.06.001 PMC2857533

[b9] Gong C., Song E., Codogno P. & Mehrpour M. The roles of BECN1 and autophagy in cancer are context dependent. Autophagy 8, 1853–1855, 10.4161/auto.21996 (2012). 22960473 10.4161/auto.21996 PMC3541303

[b10] Kotsafti A. *et al.* Autophagy and apoptosis-related genes in chronic liver disease and hepatocellular carcinoma. BMC Gastroentero. 12, 118, 10.1186/1471-230X-12-118 (2012). 10.1186/1471-230X-12-118 PMC3449193 22928777

[b11] Wang Z., Han W., Sui X., Fang Y. & Pan H. Autophagy: A novel therapeutic target for hepatocarcinoma (Review). Oncol Lett. 7, 1345–1351, 10.3892/ol.2014.1916 (2014). 24765136 10.3892/ol.2014.1916 PMC3997714

[b12] Seglen P. O. & Gordon P. B. 3-Methyladenine: specific inhibitor of autophagic/lysosomal protein degradation in isolated rat hepatocytes. Proc Natl Acad Sci U S A. 79, 1889–1892 (1982). 6952238 10.1073/pnas.79.6.1889 PMC346086

[b13] Klionsky D. J., Elazar Z., Seglen P. O. & Rubinsztein D. C. Does bafilomycin A1 block the fusion of autophagosomes with lysosomes? Autophagy 4, 849–850 (2008). 18758232 10.4161/auto.6845

[b14] Briceno E., Calderon A. & Sotelo J. Institutional experience with chloroquine as an adjuvant to the therapy for glioblastoma multiforme. Surg Neurol. 67, 388–391, 10.1016/j.surneu.2006.08.080 (2007). 17350410 10.1016/j.surneu.2006.08.080

[b15] Mousavi S. A., Brech A., Berg T. & Kjeken R. Phosphoinositide 3-kinase regulates maturation of lysosomes in rat hepatocytes. Biochem J. 372, 861–869, 10.1042/BJ20021136 (2003). 12646047 10.1042/BJ20021136 PMC1223449

[b16] Palmeira-Dos-Santos C. *et al.* Comparative study of autophagy inhibition by 3MA and CQ on Cytarabine-induced death of leukaemia cells. J Cancer Res Clin Oncol. 10.1007/s00432-014-1640-4 (2014). 10.1007/s00432-014-1640-4 PMC11824056 24659340

[b17] Xi G. *et al.* Autophagy inhibition promotes paclitaxel-induced apoptosis in cancer cells. Cancer Lett. 307, 141–148, 10.1016/j.canlet.2011.03.026 (2011). 21511395 10.1016/j.canlet.2011.03.026

[b18] Shingu T. *et al.* Inhibition of autophagy at a late stage enhances imatinib-induced cytotoxicity in human malignant glioma cells. Int J Cancer. 124, 1060–1071, 10.1002/ijc.24030 (2009). 19048625 10.1002/ijc.24030

[b19] Song J. *et al.* Hypoxia-induced autophagy contributes to the chemoresistance of hepatocellular carcinoma cells. Autophagy 5, 1131–1144 (2009). 19786832 10.4161/auto.5.8.9996

[b20] Mathew R., Karantza-Wadsworth V. & White E. Role of autophagy in cancer. Nature reviews. Cancer 7, 961–967, 10.1038/nrc2254 (2007). 10.1038/nrc2254 PMC2866167 17972889

[b21] Zhang H. *et al.* Mitochondrial autophagy is an HIF-1-dependent adaptive metabolic response to hypoxia. J Biol Chem. 283, 10892–10903, 10.1074/jbc.M800102200 (2008). 18281291 10.1074/jbc.M800102200 PMC2447655

[b22] Young J. E. & La Spada A. R. Development of selective nutrient deprivation as a system to study autophagy induction and regulation in neurons. Autophagy 5, 555–557 (2009). 19363305 10.4161/auto.5.4.8389

[b23] Ye Z. *et al.* c-Jun N-terminal kinase - c-Jun pathway transactivates Bim to promote osteoarthritis. Can J Physiol Pharmacol. 92, 132–139, 10.1139/cjpp-2013-0228 (2014). 24502636 10.1139/cjpp-2013-0228

[b24] Ramjaun A. R., Tomlinson S., Eddaoudi A. & Downward J. Upregulation of two BH3-only proteins, Bmf and Bim, during TGF beta-induced apoptosis. Oncogene 26, 970–981, 10.1038/sj.onc.1209852 (2007). 16909112 10.1038/sj.onc.1209852

[b25] Hu F. *et al.* Blocking autophagy enhances the apoptosis effect of bufalin on human hepatocellular carcinoma cells through endoplasmic reticulum stress and JNK activation. Apoptosis 19, 210–223, 10.1007/s10495-013-0914-7 (2014). 24114361 10.1007/s10495-013-0914-7

[b26] Suzuki H. I., Kiyono K. & Miyazono K. Regulation of autophagy by transforming growth factor-beta (TGF-beta) signaling. Autophagy 6, 645–647, 10.4161/auto.6.5.12046 (2010). 20458184 10.4161/auto.6.5.12046

[b27] Cosse J. P. & Michiels C. Tumour hypoxia affects the responsiveness of cancer cells to chemotherapy and promotes cancer progression. Anticancer Agents Med Chem. 8, 790–797 (2008). 18855580 10.2174/187152008785914798

[b28] Zhou J., Schmid T., Schnitzer S. & Brune B. Tumor hypoxia and cancer progression. Cancer Lett. 237, 10–21, 10.1016/j.canlet.2005.05.028 (2006). 16002209 10.1016/j.canlet.2005.05.028

[b29] Shannon A. M., Bouchier-Hayes D. J., Condron C. M. & Toomey D. Tumour hypoxia, chemotherapeutic resistance and hypoxia-related therapies. Cancer Treat Rev. 29, 297-307 (2003). 12927570 10.1016/s0305-7372(03)00003-3

[b30] Ma Y., Dai H., Kong X. & Wang L. Impact of thawing on reference gene expression stability in renal cell carcinoma samples. Diagn Mol Pathol. 21, 157–163, 10.1097/PDM.0b013e31824d3435 (2012). 22847160 10.1097/PDM.0b013e31824d3435

