# Supplementary material for: Autophagy inhibits chemotherapy-induced apoptosis through downregulating Bad and Bim in hepatocellular carcinoma cells
Source: Sci Rep. 2014 Jun 20;4:5382. doi: 10.1038/srep05382 (PMC4064348; doi:10.1038/srep05382)
Supplement: Supplementary Information — Dataset 1 [file srep05382-s1.doc]

**Autophagy inhibits chemotherapy-induced apoptosis through downregulating Bad and Bim in hepatocellular carcinoma cells**

Yan Zhou^1^, Kai Sun^1, 2^, Yi Ma^3^, Haozheng Yang^1^, Yuanliang Zhang^4^, Xianming Kong^1*^, Lixin Wei^1, 2*^

1 Medical Sciences Research Center, Ren Ji Hospital, School of Medicine, Shanghai Jiao Tong University, Shanghai 200127, China

2 Tumor Immunology and Gene Therapy Center, Eastern Hepatobiliary Surgery Hospital, The Second Military Medical University, Shanghai 200438, China

3 Department of Biobank, Ren Ji Hospital, School of Medicine, Shanghai Jiao Tong University, Shanghai 200127, China

4 Shanghai Institute of Hematology, Ruijin Hospital, School of Medicine, Shanghai Jiaotong University, Shanghai 200025, China

***Correspondence authors:**

1. Xianming Kong, Medical Sciences Research Center, Ren Ji Hospital, School of Medicine, Shanghai Jiao Tong University, 1630 Dongfang Road, Shanghai 200127, China. Tel.:+86-21-68383636. E-mail: [xianmingk@163.com](mailto:xianmingk@163.com)

2. Lixin Wei, Medical Sciences Research Center, Ren Ji Hospital, School of Medicine, Shanghai Jiao Tong University, 1630 Dongfang Road, Shanghai 200127, China. Tel.:+86-21-68383639; Fax: +86-21-65566349; E-mail: [weilixin@renji.com](mailto:weilixin@renji.com)

**
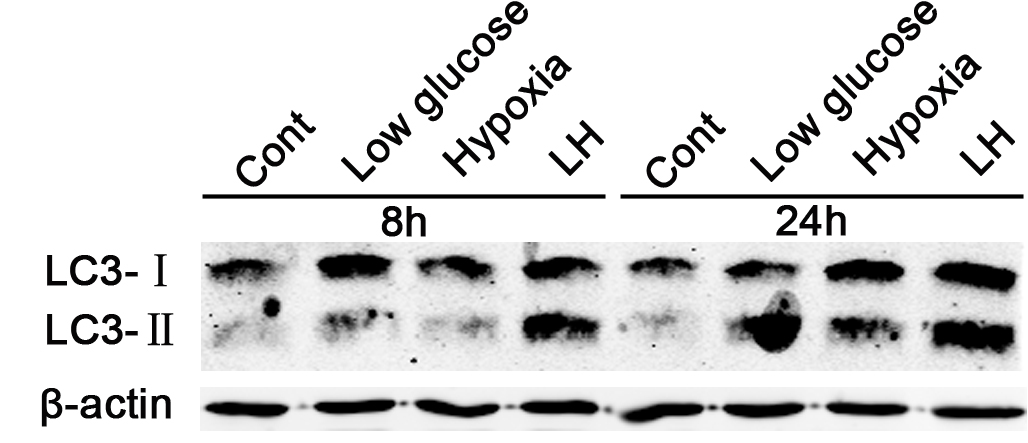
**

**Supplementary Figure 1 Autophagy is induced by the combination of low glucose and hypoxia treatments.** SMMC-7721 cells were incubated under low-glucose or hypoxia or LH condition for 8 h and 24h. The whole-cell lysates were subjected to western blot analysis, and the experiments were repeated at least three times.
